# In Silico Identification of a BRCA1:miR-29:DNMT3 Axis Involved in the Control of Hormone Receptors in BRCA1-Associated Breast Cancers

**DOI:** 10.3390/ijms24129916

**Published:** 2023-06-08

**Authors:** Manuela Santarosa, Davide Baldazzi, Michela Armellin, Roberta Maestro

**Affiliations:** Unit of Oncogenetics and Functional Oncogenomics, CRO Aviano, National Cancer Institute, IRCCS, 33081 Aviano, Italy; davide.baldazzi@cro.it (D.B.); marmellin@cro.it (M.A.); rmaestro@cro.it (R.M.)

**Keywords:** breast cancer, BRCA1, microRNA, miR-29c, miR-29b, DNMT3A, DNMT3B, DNA methylation, estrogen receptor, progesterone receptor

## Abstract

Germline inactivating mutations in the BRCA1 gene lead to an increased lifetime risk of ovarian and breast cancer (BC). Most BRCA1-associated BC are triple-negative tumors (TNBC), aggressive forms of BC characterized by a lack of expression of estrogen and progesterone hormone receptors (HR) and HER2. How BRCA1 inactivation may favor the development of such a specific BC phenotype remains to be elucidated. To address this question, we focused on the role of miRNAs and their networks in mediating BRCA1 functions. miRNA, mRNA, and methylation data were retrieved from the BRCA cohort of the TCGA project. The cohort was divided into a discovery set (Hi-TCGA) and a validation set (GA-TCGA) based on the platform used for miRNA analyses. The METABRIC, GSE81002, and GSE59248 studies were used as additional validation data sets. BCs were differentiated into BRCA1-like and non-BRCA1-like based on an established signature of BRCA1 pathway inactivation. Differential expression of miRNAs, gene enrichment analysis, functional annotation, and methylation correlation analyses were performed. The miRNAs downregulated in BRCA1-associated BC were identified by comparing the miRNome of BRCA1-like with non-BRCA1-like tumors from the Hi-TCGA discovery cohort. miRNAs:gene-target anticorrelation analyses were then performed. The target genes of miRNAs downregulated in the Hi-TCGA series were enriched in the BRCA1-like tumors from the GA-TCGA and METABRIC validation data sets. Functional annotation of these genes revealed an over-representation of several biological processes ascribable to BRCA1 activity. The enrichment of genes related to DNA methylation was particularly intriguing, as this is an aspect of BRCA1 functions that has been poorly explored. We then focused on the miR-29:DNA methyltransferase network and showed that the miR-29 family, which was downregulated in BRCA1-like tumors, was associated with poor prognosis in these BCs and inversely correlated with the expression of the DNA methyltransferases DNMT3A and DNMT3B. This, in turn, correlated with the methylation extent of the promoter of HR genes. These results suggest that BRCA1 may control the expression of HR via a miR-29:DNMT3:HR axis and that disruption of this network may contribute to the receptor negative phenotype of tumors with dysfunctional BRCA1.

## 1. Introduction

Patients carrying inactivating BRCA1 germline mutations have an increased lifetime risk of developing breast and ovarian cancer [1]. Specifically, BRCA1 germline mutations predispose to the occurrence of triple-negative breast cancer (TNBC), an aggressive subtype of breast cancer (BC) that is negative for the expression of the hormone receptors (HR), ERα and PR, and HER2 [2,3].

In addition to germline mutations, which account for approximately 5–10% of all BCs, a subset of sporadic cancers, most of the TNBC subtype, have features of dysfunctional BRCA1 [4,5,6]. Approximately 10% exhibit aberrant methylation of the BRCA1 promoter, a feature closely associated with the downregulation of BRCA1 gene transcripts and loss of heterozygosity at the BRCA1 locus [7,8,9]. This fraction of tumors shows hallmarks of dysfunctional BRCA1 characteristics of hereditary BRCA1 tumors, such as the absence of HR, amplification of MYC, and an unchanged ERBB2 gene (encoding the HER2 receptor) [10,11]. Finally, patterns of genomic alterations typical of familial BRCA1 tumors are detected in an additional fraction of sporadic BCs devoid of evident BRCA1 gene or expression alterations [11,12]. Thus, in addition to hereditary BRCA1 tumors, a substantial proportion of sporadic BCs may be considered BRCA1-associated since they rely on dysfunctional BRCA1 as a driving force. To better identify these tumors, Chen and coworkers developed a specific signature that they used to define a group of BRCA1-like tumors [5].

The large nuclear BRCA1 protein acts as a guardian of chromosome integrity through various functions that ensure the assembly and activity of macromolecular complexes involved in DNA double-strand break repair [13] and mitotic and replication control [14]. Tumors with deleterious mutations in the BRCA1 gene and hence impaired DNA repair capacity are highly sensitive to inter-strand crosslinking agents (platinum or alkylating agents), topoisomerase II inhibitors (anthracyclines), and PARP inhibitors [15,16,17].

Increasing evidence indicates that BRCA1 also plays a central role in regulating the transcription and processing of RNA [18,19], including non-coding microRNAs (miRNAs) [20]. miRNAs are important regulators of gene expression by inhibiting the translation of target mRNAs, mostly by binding to their 3′UTR region [21,22]. BRCA1 is involved in both the induction and repression of miRNA transcription and maturation through the binding to DROSHA [20,23,24,25]. Indeed, several miRNAs have been identified as deregulated in BRCA1/2-associated BCs [26,27], but how the interplay between BRCA1 and miRNAs may promote the development of the BRCA1-like/TNBC subtype remains unclear.

To identify miRNA:mRNA circuits involved in the induction of a BRCA1-like phenotype, we interrogated publicly available transcriptional profiling data. This approach allowed us to unveil a particular interplay between the miR-29 family of miRNAs, DNA methyltransferases, and HR.

## 2. Results

### 2.1. Downregulated miRNAs in BRCA1-like Tumors

**Figure 1:**
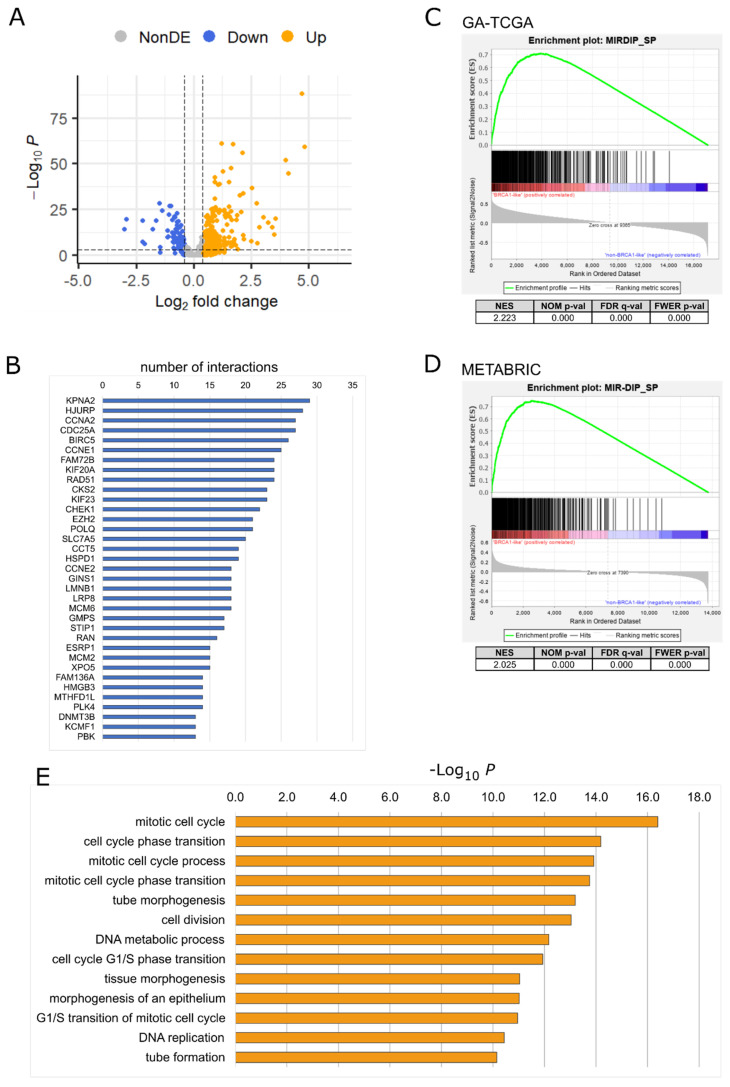
Targets and pathways affected by miRNAs differentially expressed in BRCA1-like and non-BRCA1-like cancers. (**A**) Volcano plot showing upregulated (Up, orange dots) and downregulated (Down, blue dots) miRNAs resulting from differential expression analysis of miRNA isoforms in the Hi-TCGA data set. The comparison was made between BRCA1-like and non-BRCA1-like cancers. The horizontal dashed line corresponds to the *p*-value cut-off (*p*-value < 0.0001, −Log(*p*-value) > 4). Vertical dashed lines indicate fold change cut-off values (log2FC > |0.4|). Gray dots (NonDE) represent non-differentially expressed miRNAs (R package EnhancedVolcano). (**B**) The 35 most targeted genes ranked by the number of interactions with distinct miRNAs. Interactions refer to significant anti-correlation (r < −0.35) between downregulated miRNAs and putative targets. (**C**,**D**) GSEA plots showing the enrichment of 504 miRNA targets in BRCA1-like compared with non-BRCA1-like in the GA-TCGA (**C**) and METABRIC (**D**) validation series. Normalized enrichment score (NES), nominal *p*-value (NOM *p*-value), false discovery rate (FDR), and familywise-error rate (FWER *p*-value) are reported. (**E**) Results of gene ontology analysis for the most significantly enriched biological processes associated with the 504 targets of downregulated miRNAs (full data are provided in Supplemental Table S3).

To address the hypothesis of a role for BRCA1:miRNA networks in the development of the BRCA1-like/TNBC subtype, microRNA expression data were retrieved from the TCGA-BRCA project (https://www.cancer.gov/tcga) [28]. To capture all cases with a putative dysfunctional BRCA1, we used the classification into BRCA1-like and non-BRCA1-like cases proposed by Chen and coworkers [5]. We used the TCGA BC miRNA isoform data generated by Hiseq Illumina technology (Hi-TCGA data set) as the discovery data set. This consists of 554 patients, 161 BRCA1-like, and 393 non-BRCA1-like. Differential expression analysis showed that 57 miRNAs were downregulated and 105 were upregulated in BRCA1-like compared with non-BRCA1-like samples (log2FC > |0.4|, padj < 0.00001; Figure 1A and Supplemental Table S1).

To uncover genes whose overexpression, as a consequence of the alleviation from miRNAs-mediated inhibition, may promote tumorigenic properties, we focused on downregulated miRNAs and queried the miRDIP platform (version 5.0.2.3, https://ophid.utoronto.ca/mirDIP/; accessed on 20 April 2022). This is a large database for predicting miRNA:gene target pairs based on 3′UTR pairing [29]. The 57 downregulated miRNAs were predicted to target 4236 genes with high confidence (very high score). We assumed that if a miRNA acts as a negative regulator of a given mRNA, its expression levels should be somehow anticorrelated. Therefore, we retrieved the RNAseq data for the same cases of the Hi-TCGA BC series and performed anticorrelation analyses with miRNA expression. Using a correlation threshold R < −0.35 as a cutoff, 2215 interactions were observed between 41 of the 57 miRNAs used as input and 504 genes (Supplemental Table S2). Some genes were targeted by nearly 30 miRNAs (Figure 1B).

After having identified these anticorrelated miRNA:gene pairs using the Hi-TCGA discovery cohort, we sought to verify whether the genes potentially targeted by the miRNAs downregulated in BRCA1-like cases were enriched in BRCA1-like BC. We then performed gene set enrichment analysis (GSEA) on the mRNA data obtained from two independent data sets: the first, consisting of 76 BRCA1-like and 149 non-BRCA1-like BCs, was generated by the TCGA consortium using a genome analyzer sequencing platform to produce miRNA data (GA-TCGA); the second data set was obtained from the METABRIC project and included 215 BRCA1-like and 1068 non-BRCA1-like BCs [30]. The GSEA revealed that the 504 genes potentially targeted by downregulated miRNAs were indeed significantly enriched in BRCA1-like samples of both validation series (Figure 1C,D), supporting the hypothesis of a BRCA1-mediated control over these genes via miRNAs.

Functional annotation by overrepresentation analysis (ORA) of the genes targeted by the downregulated miRNAs revealed that many of the over-represented biological processes of gene ontology (GO) were known to be controlled by BRCA1, such as cell cycle and cell division and DNA metabolic processes. Among the genes belonging to these processes, we noticed the involvement of the DNA methyltransferases, including DNMT3A and DNMT3B (Figure 1E, Supplemental Table S3; Supplemental Figure S1). The two DNA methyltransferases catalyze the methylation of genomic DNA to establish new DNA methylation patterns during embryogenesis [31] and in cancer [32,33].

### 2.2. miR-29s:DNMT3A-DNMT3B Network Is Involved in BRCA1-like Tumors

**Figure 2:**
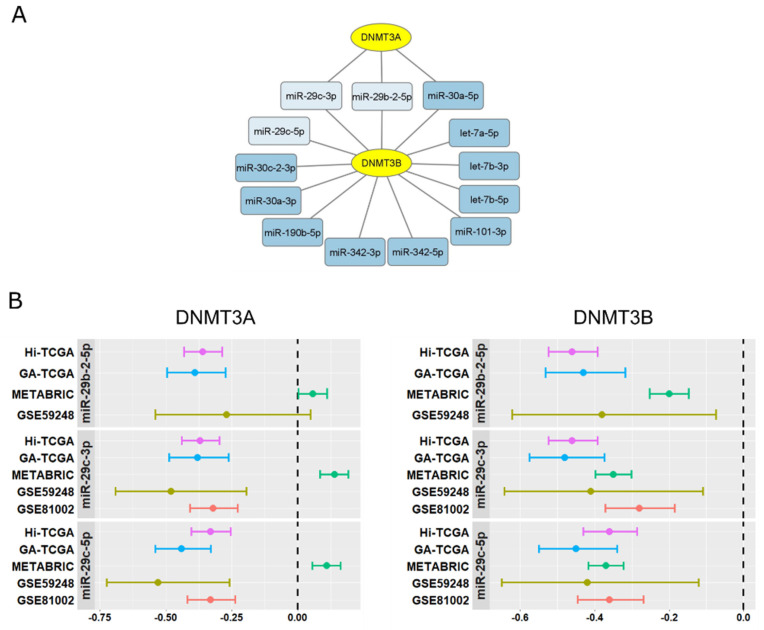
miR-29 family members target the DNMT3A and DNMT3B. (**A**) Network of miRNAs (blue rectangles) targeting DNA methyltransferases DNMT3A and DNMT3B (yellow ellipses). miR-29 family members are evidenced in light blue. (**B**) Forest plots show the correlation between miR-29b-2-5p, miR-29c-3p, miR-29c-5p, and either DNMT3A (left panels) or DNMT3B (right panels) in the five cohorts described in the text. The dots indicate the Spearman coefficient and the error bars the 95% confidence intervals of each study. The dashed line represents the Spearman coefficient equal to 0.

Correlation analysis revealed an inverse relationship between DNMT3A and DNMT3B and a limited number of miRNAs downregulated in BRCA1-like tumors. The miR-29 family was strongly represented (Figure 2A; Supplemental Table S2) and capable of affecting both DNMT3A and DNMT3B. Indeed, the anti-correlation between miR-29b-2-5p, miR-29c-3p, and miR-29c-5p transcripts and DNMT3A and DNMT3B levels was demonstrated not only in the Hi-TCGA discovery series but also in the GA-TCGA validation data set and in two other cohorts containing data for both miRNAs and coding mRNA, GSE59248 (39 patients, [34]) and GSE81002 (377 patients, [35]), (Figure 2B). DNMT3A did not result in being anticorrelated with the three miRNAs in the METABRIC series; nevertheless, a high anti-correlation between miR-29s and DNMT3B was confirmed in this cohort (Figure 2B).

**Figure 3:**
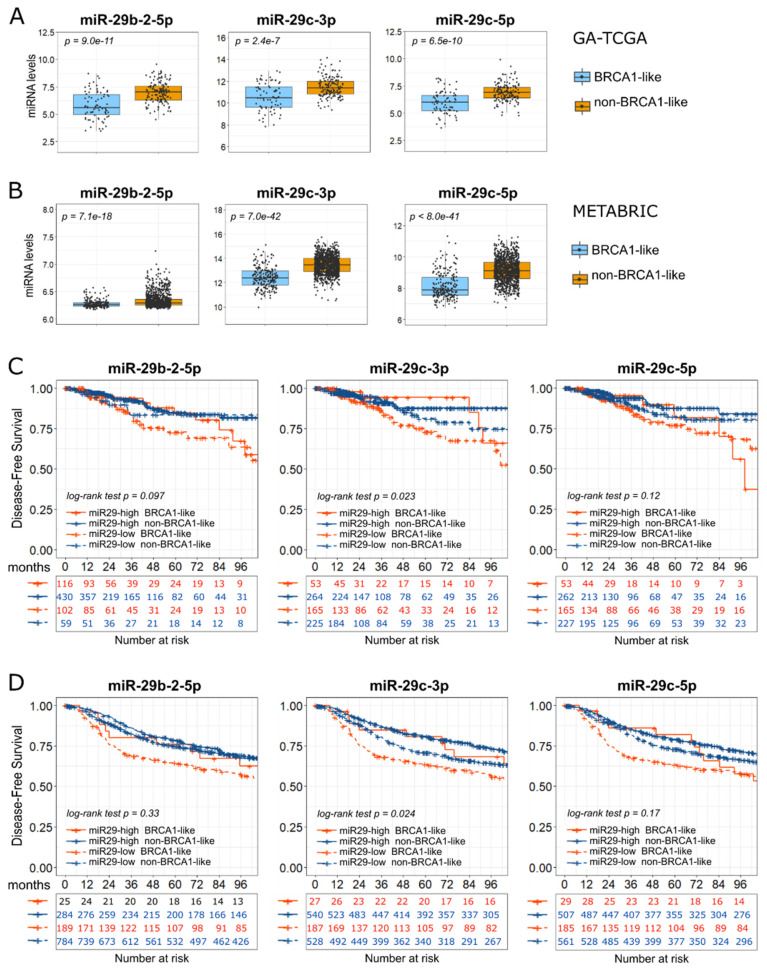
Role of miR-29 family members in BRCA1-like BCs. (**A**) Boxplots show the levels of miR-29 family members (log2 reads-per-million-miRNA-mapped), from the GA-TCGA series, in the BRCA1-like (76) and non-BRCA1-like (149) cases. (**B**) miR-29s levels (log2 normalized value) from METABRIC series according to BRCA1 status: 215 cases were BRCA1-like, 1068 were non-BRCA1-like. Boxplots show median values and first and third quartiles; whisker lengths correspond to 1.5 times the interquartile range. Statistical differences were determined with the Welch two-sample *t*-test. (**C**,**D**) Kaplan–Meier survival curves representing disease-free probability according to the levels of miR-29 family members and BRCA1 status: (**C**) TCGA cohort consisting of Hi-TCGA and GA-TCGA data; (**D**) METABRIC data. Samples were divided into “high” (solid lines) and “low” (dashed lines) miRNA-expressing samples according to the best cutpoint (as described in Materials and Methods). Samples were further split into BRCA1-like (orange lines) and non-BRCA1-like (blue lines) samples. A log-rank test was used to determine the differences between curves. The numbers under the graphs represent patients at risk at a specific time point (months). Survival curves are truncated at 100 months.

Therefore, we investigated the role of miR-29s in BC and performed a more detailed analysis of the miR-29s:DNMT3A-DNMT3B network. First, we confirmed the downregulation of miR-29b-2-5p, miR-29c-3p, and miR-29c-5p in BRCA1-like BCs in both GA-TCGA and METABRIC validation sets (Figure 3A,B).

**Figure 4:**
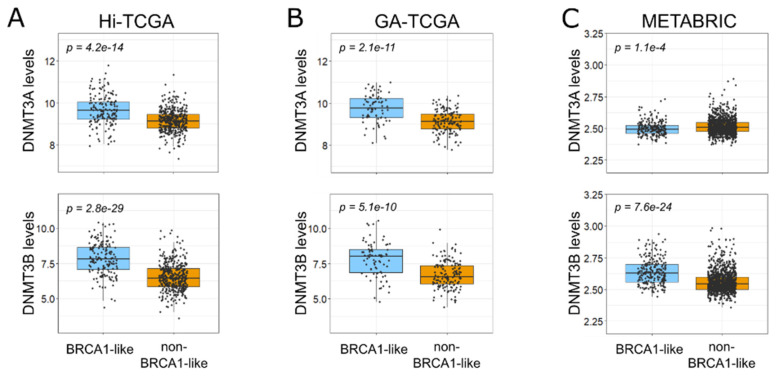
Different levels of DNMT3A/DNMT3B transcripts according to BRCA1 status. (**A**,**B**) DNMT3A (upper panels) and DNMT3B (lower panels) levels, expressed as log2 RSEM-normalized value, in the BRCA1-like and non-BRCA1-like BC samples of the Hi-TCGA series ((**A**) 161 and 393 patients, respectively) and GA-TCGA ((**B**) 76 and 149 patients, respectively). (**C**) DNMT3A (upper panels) and DNMT3B (lower panels) levels, expressed as log2-normalized value, in the BRCA1-like (215) and non-BRCA1-like (1068) BC samples from the METABRIC cohort. Boxplots show median values and first and third quartiles; whisker lengths correspond to 1.5 times the interquartile range. Statistical differences were determined with the Welch two-sample *t*-test.

We then examined the prognostic role of miR-29 members in both TCGA and METABRIC cohorts. Due to the limited number of BRCA1-like samples with high miR-29s levels, we pooled Hi-TCGA and GA-TCGA data after having calculated miR-29s z-scores. Intriguingly, the BRCA1-like group with low levels of the three miR-29s demonstrated a trend toward the worst disease-free survival (DFS) probability in both the TCGA (Figure 3C) and METABRIC (Figure 3D) data sets. The differences between groups reached statistical significance in the case of miR-29c-3p, the most highly expressed of the three miRNAs. The clinical relevance of miR-29s may involve DNMT3A and DNMT3B, which were indeed upregulated in both the Hi-TCGA discovery group and the GA-TCGA validation cohort (Figure 4A,B). Consistent with the correlation data, in the METABRIC series, only DNMT3B was significantly increased in BRCA1-like cancers compared with non-BRCA1-like samples (Figure 4C).

**Figure 5:**
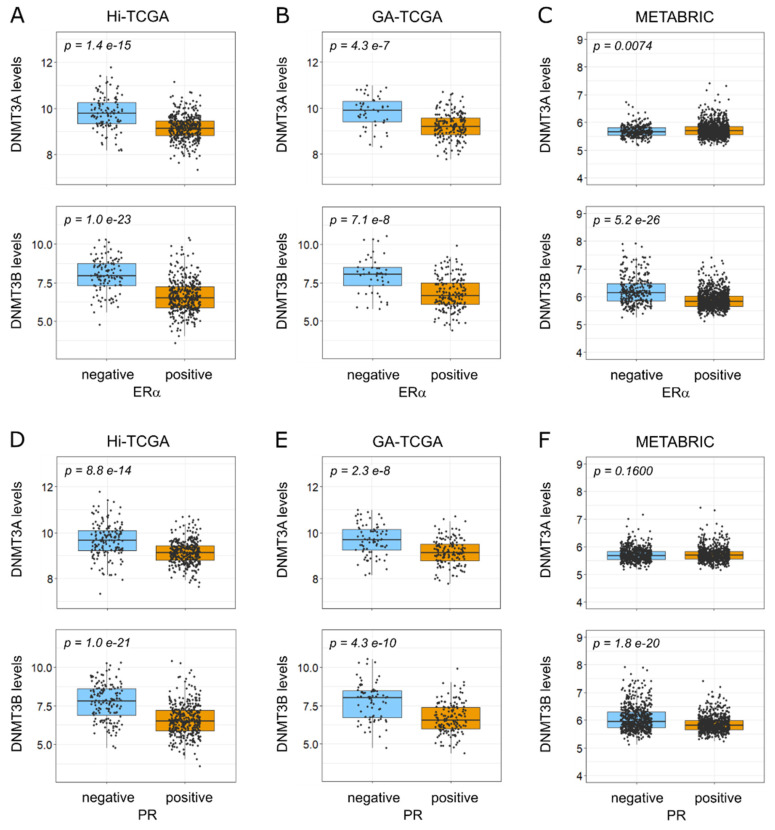
Differential distribution of DNMT3A/DNMT3B levels according to hormone receptor status. (**A**–**C**) DNMT3A (upper panels) and DNMT3B (lower panels) levels according to ERα receptor sample status from Hi-TCGA ((**A**) 112 negative and 406 positive cases), GA-TCGA ((**B**) 46 negatives and 177 positives), and METABRIC ((**C**) 300 positives and 983 negatives). (**D**–**F**) as in (**A**–**C**), DNMT3s levels according to PR status: (**D**) Hi-TCGA series (149 negative and 368 positive samples); (**E**) GA-TCGA series (77 negatives and 146 positives); (**F**) METABRIC series (608 negatives and 675 positives). Boxplots show median values and first and third quartiles; whisker lengths correspond to 1.5 times the interquartile range. Statistical differences were determined with the Welch two-sample *t*-test.

Interestingly, DNMT3s expression is associated with typical features of BRCA1-like samples, such as negativity for HR. Indeed, DNMT3B transcripts were significantly and markedly higher in samples classified as negative for either estrogen receptor-α (ERα) or progesterone receptor (PR) in both the Hi-TCGA discovery cohort and the GA-TCGA and METABRIC validation series (Figure 5A,F, lower panels). DNMT3A transcripts were significantly higher in hormone receptor-negative samples from both Hi-TCGA and GA-TCGA (Figure 5A,B,D,E, upper panels), but not in the METABRIC series (Figure 5C,F), consistent with the lack of correlation with miR-29 family members and the absence of upregulation in BRCA1-like tumors in this cohort. We further examined the GSE81002 and GSE59248 series: DNMT3A and DNMT3B levels were higher in ERα-negative than in positive cases in both series (Supplemental Figures S1 and S2A) and in PR-negative samples in GSE59248 (Supplemental Figure S1B; the GSE81002 project did not provide PR status).

### 2.3. DNA Methyltransferases Affect ESR1 and PGR DNA Methylation in BRCA1-like Samples

The data reported so far suggest a potential form of control over DNMT3s by BRCA1 via miR-29 family members and the association of DNMT3s expression with the absence of HR.

**Figure 6:**
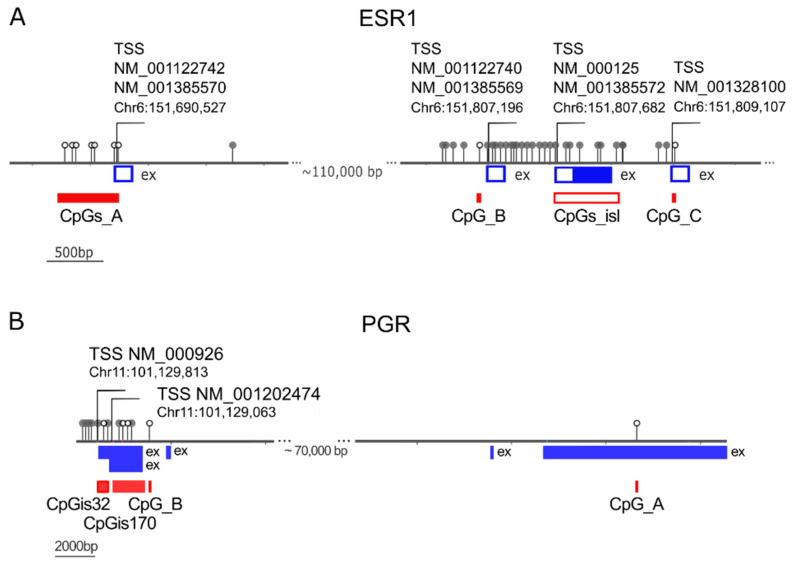
Methylation of ESR1 and PGR genes. (**A**) Map of a portion of the genomic region of ESR1 (GRCh38/hg38, chr6:151,690,000–151,810,000) showing distinct transcription start sites (TSS), the exons mapping in the reported DNA segment; the CpGs evaluated with the Illumina Infinium Human DNA Methylation 450 platform. The CpGs not associated with ESR1 expression are depicted in gray, those significantly associated with ESR1 gene expression are shown as empty circles and are highlighted by filled red rectangles (CpGs_A, CpG_B, and CpG_C regions). The CpG island region (CpGs_isl) is shown as an empty red rectangle. (**B**) Map of the PGR genomic region shown as the reverse complement sequence (GRCh38/hg38, chr11: 101,129,813–101,029,624). Two TSSs are shown, NM_000926 and NM_001202474, encoding isoform B and isoform A, respectively. The CpGs Inversely associated with PGR gene expression are shown as empty circles and highlighted by the filled red rectangles of CpG_B and CpG_A and the two CpG islands (CpGis32 and CpGis170). The other CpGs in the Illumina Infinium Human DNA Methylation 450 platform are drawn as gray circles. Genomic coordinates of the CpGs mapping on the ESR1 and PGR genes are reported in Supplemental Tables S4 and S5.

Interestingly, genes encoding ERα (ESR1 gene) and PR (PGR gene), which are weakly expressed in BRCA1-associated tumors [36], have been reported to be downregulated by methylation of promoter CpGs [37,38,39,40]. Therefore, we sought to determine whether the upregulation of DNA methyltransferases was associated with promoter methylation of ESR1 and PGR genes. To investigate this hypothesis, we obtained from the TCGA-BRCA project the methylation data of the genomic regions where ESR1 and PGR were mapped (Figure 6A,B). The ESR1 gene spans more than 400 Kbp, includes 8 coding exons and, through the use of alternative promoters and splicing, forms dozens of transcript variants (RefSeq: NCBI Reference Sequence Database), which in turn are translated into at least 4 isoforms [41]. The PGR gene encodes two major isoforms, isoform A (PRA) and B (PRB), which are derived from two alternative promoters and two different transcription start sites (TSS) [42].

The TCGA methylation data consisted of β-values, the estimates of methylation levels, for 63 probes along the ESR1 gene and 17 probes for the PGR gene.

To determine which methylated CpGs might be relevant to control ESR1 and PGR transcript levels, we used data from Hi-TCGA patients (433 samples) and categorized hormone-positive and -negative samples based on the pit of the bimodal distribution of ESR1 and PGR log2RSEM-normalized counts (Supplemental Figure S3). A significant fold change >1.5 of CpG β-values in HR negative over positive samples was used to select CpGs whose methylation inhibits ESR1 and PGR expression. A limited number of CpG probes (Supplemental Table S4) met these criteria for the ESR1 gene: seven CpG probes (CpGs_A; Figure 6A) located near the transcription start sites (TSSs) of RefSeq NM_001122740 and NM_001385570 (variants 4 and 10, respectively) located far upstream of the canonical NM_000125 variant 1; a CpG (CpG_B) in the region of promoter B [38] near the TSSs of NM_001122740 and NM_001385569 (variants 2 and 9, respectively) and a CpG (CpG_C) close to TSS of NM_001328100 (variant 7). The available data for the CpG island were very limited and included only 5 of the 105 CpGs reported in the CpG islands track of the UCSC genome browser (https://genome.ucsc.edu/; accessed on 1 December 2022). These CpGs were only weakly methylated and appeared to scarcely affect ESR1 expression in the BC series of TCGA.

In the case of the PGR gene, two CpGs tested by TCGA probes (Supplemental Table S5), CpG_A and CpG_B, showed β-values FC > 1.5 and were selected for subsequent analyses (Figure 6B). In addition, the methylation of CpG islands seems to control PGR expression, even though the number of probes was limited (3 of 32 for CpGs-island 32 and 4 of 170 for CpGs-island170).

**Figure 7:**
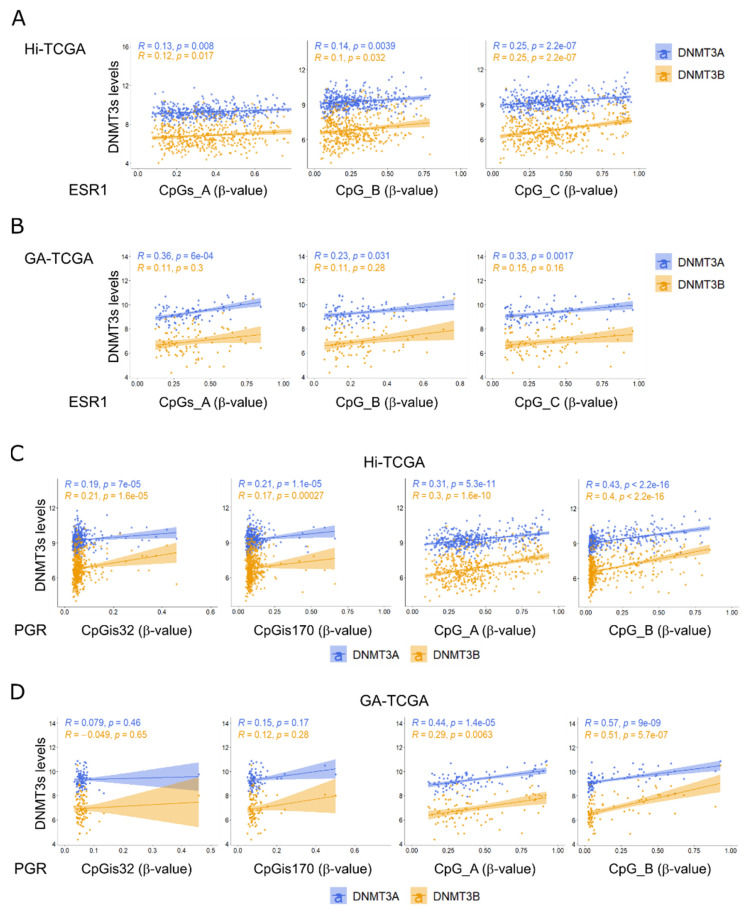
Correlation between DNMT3A and DNMT3B expression and methylation of ESR1 and PGR genes. (**A**,**B**) Correlation between either DNMT3A (light blue dots and line) or DNMT3B (orange dots and line) levels (log2RSEM-normalized values) and the average β-values of CpGs_A, CpG_B, and CpG_C in the ESR1 gene in the Hi-TCGA (**A**) and GA-TCGA (**B**) series. (**C**,**D**) Correlation plots between either DNMT3A (light blue dots and line) or DNMT3B (orange dots and line) and the β-values of CpGis32, CpGis170, CpG_A, and CpG_B in the PGR gene. Spearman correlation coefficients R and *p*-value are indicated in the same color as the corresponding DNMT3 gene. Genomic coordinates of the CpGs mapping on the ESR1 and PGR genes are reported in Supplemental Tables S4 and S5.

Next, we wanted to test whether DNMT3A and DNMT3B might have a role in the methylation of the selected CpGs in the ESR1 and PGR regions. To this end, we examined the correlation between the average β-values of the 7 CpGs in CpGs_A, the single CpG_B, and CpG_C β-values of ESR1 and the expression levels of DNMT3A and DNMT3B. Only CpG_C significantly correlated with DNMT3A in both the Hi-TCGA (Figure 7A) and GA-TCGA (90 samples; Figure 7B) series. The low levels of CpG methylation of the CpG island did not correlate with DNMT3s expression in both Hi-TCGA and GA-TCGA cohorts (Supplemental Figure S4).

Interestingly, both CpG_A and CpG_B of the PGR gene correlated markedly and significantly with both DNMT3A and DNMT3B in the Hi-TCGA and GA-TCGA cohorts (Figure 7C,D). The levels of DNMT3A and DNMT3B transcripts were not associated with the methylation of CpG islands, which were barely methylated in either gene (Figure 7C,D).

Thus, DNMT3A and DNMT3B were associated with the methylation of a number of CpGs that control the expression of HR genes, particularly of PGR.

**Figure 8:**
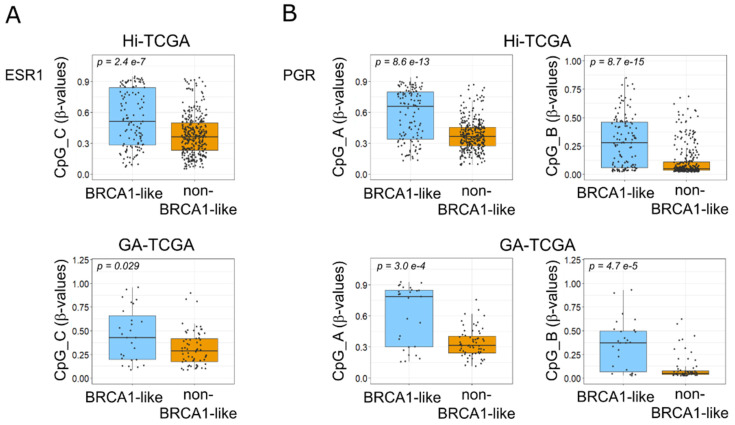
Methylation of specific CpGs in ESR1 and PGR genes according to BRCA1 status. Comparison of β-values of ESR1 CpG_C (**A**) and PGR CpG_A and CpGs_B (**B**) in BRCA1-like compared with non-BRCA1-like BCs. Upper panels show data from the Hi-TCGA series (121 BRCA1-like and 312 non-BRCA1-like cases); lower panels report data from the GA-TCGA cohort (27 BRCA1-like and 63 non-BRCA1-like cases). Boxplots show median values and first and third quartiles; whisker lengths correspond to 1.5 times the interquartile range. Statistical differences were determined with the Welch two-sample *t*-test.

Finally, to come full circle, we showed that the methylation levels of CpGs_C in the ESR1 gene and CpG_A and CpG_B in the PGR gene were indeed higher in BRCA1-like compared with BRCA1-non-like tumors in both Hi-TCGA and GA-TCGA series (Figure 8A,B).

## 3. Discussion

Inheritance of a germline mutation of the BRCA1 gene predisposes with high penetrance to breast, ovarian, and other forms of epithelial carcinoma [43]. Interestingly, the vast majority of breast cancers that occur in BRCA1 mutation carriers have a TNBC phenotype that prevents patients from being treated with therapies targeting hormone receptor pathways [3]. Despite much progress in elucidating how dysfunctional BRCA1 causes the occurrence of such specific BCs, we still do not have a complete picture. It is known that BRCA1 can both inhibit ERα signaling and induce ESR1 gene expression by binding coactivators such as BRD7 and OCT1 or resolving R-loops in the enhancer region upstream of the ESR1 gene, allowing ESR1 transcription [44,45,46,47,48]. In addition, it can regulate the PR pathway indirectly via ESR1, which induces transcription of the PGR gene, and also interact directly with PRA and PRB [42,49,50].

Here, we propose that the downregulation of specific miRNAs, such as miR-29 family members, contributes to the lack of HR expression in BRCA1-like tumors.

There is increasing evidence that BRCA1 controls the expression of miRNAs through various mechanisms: transcriptional repression, activation, or by controlling miRNA biogenesis via the DROSHA microprocessor complex [20,23,25]. Either way, in the present analyses, we observed several miRNAs deregulated in BRCA1-like tumors. The targets of these miRNAs were involved in pathways known to be influenced by BRCA1 mutations, namely cell cycle, cell division, and DNA replication. Of note, the DNA methyltransferases DNMT3A and DNMT3B emerged among the genes involved in these pathways. They anticorrelated with miR-29 family members and were upregulated in BRCA1-like cancers. In vitro inhibition of these methyltransferases by miR-29 family members has already been demonstrated for several pathologies [51,52].

Although we could not demonstrate in these in silico analyses whether and how BRCA1 directly regulates miR-29 expression, a recent paper reported that BRCA1 binds cis-regulatory elements in the promoter region of miR-29b-1 (in a cluster with miR-29a on chromosome 7) and induces its expression [53]. We did not verify whether this form of control also applies to the other miR-29s, but it is worth noting that the genes encoding the most downregulated miR-29c -3p and -5p, and miR-29b-2-5p are clustered together on chromosome 1, suggesting a common regulation for these miRNAs. In this scenario, BRCA1 dysfunction could be the cause of low levels of these miR-29 family members. Interestingly, several lines of evidence suggest that miR-29s are downregulated in cancer and that their reduction is associated with a poor prognosis [54,55,56]. Here, we showed that the prognostic role of miR-29s was mainly associated with BRCA1-like samples. Indeed, low levels of these miRNAs, especially miR-29c-3p, in the BRCA1-like samples showed the worst prognosis and were associated with the short-term relapse that is typical of TNBC.

Consistent with the role of miR-29s in cancer, their targets DNMT3A and DNMT3B have been described as overexpressed in various neoplasms and associated with poor prognosis in numerous tumors, including BC [32,33,51,57,58,59,60].

DNMT3A and DNMT3B are de novo methyltransferases that transfer a methyl group to the C-5 position of the cytosine residue to establish DNA methylation [61,62]. They may control gene expression by methylating their promoters, a mechanism that has long been considered critical for gene silencing in cancer development.

Even the MIR29C and MIR29B2 regions were methylated but the extent of methylation did not correlate with either miRNA expression or with DNMTs. Curiously, methylation of CpGs 20kbp upstream of miRNAs, which Poli E et al. reported to be inversely correlated with miR-29c and miR-29b-2 expression [63], actually partially correlated with the poorly expressed strands (mir-29c-5p and miR-29b-2-5p). No correlation was observed between the methylation of CpGs in the miR-29a/miR-29b-1 locus and miRNA expression. Thus, overall, our analyses suggest that the anti-correlation between DNMT3A/3B and miR-29s is not a consequence of DNMT3A/B controlling miR-29s expression but rather the opposite. Nevertheless, we cannot rule out a negative feedback loop in which DNMTs might partially control miR-29c expression by methylating its gene.

Remarkably, the data reported here showed a correlation between DNMT3A and DNMT3B expression and negativity for ERα and PR and a direct correlation with promoter methylation levels of HR, particularly the PGR gene, suggesting a BRCA1-mediated control of these genes via the miR-29s/DNMT3A-DNMT3B axis.

In the ESR1 gene, the CpG most affected by the miR-29s/DNMT3A-DNMT3B circuit is CpG_C, which maps close to the TSS of transcripts that produce the 46 and 36 kDa ERα truncated isoforms (ERα46 and ERα36, respectively). Both isoforms have been reported to be expressed in BC, even in TNBC, and to exert opposite functions in controlling cancer progression: the best studied ERα36 is capable of activating multiple signaling pathways critical for cancer aggressiveness and metastatic potential, whereas ERα46 associates with lower grade and smaller tumors [64,65,66,67]. Further studies are needed to characterize the isoforms affected by CpG DNA methylation and to discover the role of promoter methylation/expression of these isoforms in BC progression.

In the case of PGR, DNMT3A and DNMT3B are strongly associated with methylation of the two CpGs that appear to inhibit PGR expression, even if one of them maps on an exon far from the TSS of the PGR gene. Several lines of evidence suggest that CpG methylation plays a role in controlling gene expression even when far from the promoter by altering chromatin structure and binding of transcription factors [68,69]. Importantly, the two CpGs in the PGR gene were more methylated in BRCA1-like tumors than in non-BRCA1-like tumors.

We recognize that this work has the limitations of an in silico study that showed correlations and, thus, needs further in vitro validation to confirm the results. In addition, we used the BRCA1-like and non-BRCA1-like classification as a surrogate of a categorization that considered all types of BRCA1 inactivation (e.g., mutation, methylation, small or large deletion). The fact that miR-155, a miRNA known to be repressed by BRCA1, emerged among the miRNAs overexpressed in BRCA1-like supports the efficacy of this categorization. Thus, by analyzing a series of data sets, this study identifies a potential network in which the absence of a functional BRCA1 epigenetically suppresses HR. This network is thus implicated in the development of the typical BRCA1-associated tumors that barely express ERα and PR. Furthermore, this study extends the knowledge of DNA methylation control over ESR1 gene expression in the context of BRCA1-like cancers, as reported for a limited set of CpGs in the seminal work of Archey and coworkers [70]. Interestingly, promoter methylation and lack of HR expression lead to poor prognosis and resistance to a number of anti-hormonal therapies in BC [38,40,71].

Therefore, the network described here, in which dysfunctional BRCA1 leads to the downregulation of miR-29 accompanied by an increase in DNMT3A and DNMT3B and hormone gene methylation, paves the way for the development of novel strategies to overcome endocrine resistance in BCs.

## 4. Materials and Methods

### 4.1. Data Sets

In selecting data sets for this study, we first chose those that contained mRNA and miRNA data and the BRCA1 status of the same samples. Additional data sets, even without BRCA1 status information, were selected for specific validation (see the workflow in Supplemental Figure S5).

#### 4.1.1. TCGA

TCGA data were downloaded from Broad GDAC Firehose (https://gdac.broadinstitute.org/; accessed on 7 October 2020). They included: miRNA-seq data illuminahiseq_mirnaseq-miR_isoform_expression (Hi-TCGA data set) and illuminaga_mirnaseq-miR_isoform_expression (GA-TCGA data set); RNA-seq data (illuminahiseq_rnaseqv2-RSEM_genes); DNA methylation data (humanmethylation450-within_bioassay_data_set_function-Illumina Infinium Human DNA Methylation 450 platform), and clinical data for clinicopathological features.

For miRNA isoforms, we used both the read counts from the Hi-TCGA data set and the normalized count in reads-per-million-miRNA-mapped from the Hi-TCGA and GA-TCGA data sets (TCGA miRNA pipeline at https://docs.gdc.cancer.gov/Data/Bioinformatics_Pipelines/miRNA_Pipeline/). For survival analyses, we used both the Hi-TCGA and GA-TCGA data sets. We calculated the z-score of the log2 of the normalized count in reads-per-million-miRNA-mapped for each cohort:

z-score = (miR-X sampleA − mean miR-X allSamples)/ds miR-X allSamples

Subsequently, the z-scores of the two platforms were merged.

Gene-level transcription estimates from the RNA-seq data were expressed as log2 RSEM-normalized counts. DNA methylation data report the methylation score for each CpG site expressed as β-values, which ranged from zero to one, corresponding to unmethylated and fully methylated DNA, respectively (https://docs.gdc.cancer.gov/Data/Bioinformatics_Pipelines/). We analyzed the β-values of 63 probes in the ESR1 gene region (chr6: 152,011,103–152421432; GRCh37/hg19) and of 17 probes in the PGR region (chr11: 100,905,070–101001533; GRCh37/hg19).

#### 4.1.2. METABRIC

Molecular Taxonomy of Breast Cancer International Consortium (METABRIC) data [72] were obtained from the European Genome-Phenome Archive (EGA; https://ega-archive.org/; accessed on 22 February 2021) and comprised EGAD00010000434 Normalised mRNA expression data (IlluminaHT 12 array); EGAD00010000438 Normalised miRNA expression data (Agilent ncRNA 60k); and clinicopathological features including HR status. The log2-transformed normalized data were used for the analyses.

#### 4.1.3. Other Validation Data Sets

Two independent data cohorts with miRNA, mRNA, and clinicopathological data were identified in GEO data sets (https://www.ncbi.nlm.nih.gov/gds; accessed on 18 May 2022): the GSE81002 and GSE59248 series. GSE81002 included data from the Human miRNA Microarray v14 Rev.2 and the Agilent-028004 SurePrint G3 Human GE 8 × 60K Microarray for 377 patients [35]. GSE59248 contained data from the same platform as GSE81002 for mRNA quantification and data from Agilent-031181 Unrestricted_Human_miRNA_V16.0_Microarray 030, 840, for 39 samples [34]. Since these data sets contained data for multiple probe sets, we collapsed probes into miRNA by selecting the probe with the highest average expression [73].

### 4.2. Differential Expression Analysis

We performed differential expression analysis using the Hi-TCGA data set, which includes 161 BRCA1-like and 393 non-BRCA1-like samples. Read counts of miRNA isoforms were used for the analysis, which was performed using the DESeq2 R package version 1.36.0 [74]. miRNAs with log2FC > |0.4|, BH-adjusted *p* values (padj) <0.00001, and base mean >5 normalized counts were considered differentially expressed.

### 4.3. GSEA Analyses and Gene Ontology (GO) Enrichment Analysis

Gene set enrichment analysis (GSEA) was performed using GSEA version 4.0.3 software downloaded from https://www.gsea-msigdb.org/gsea/index.jsp [75,76]. Weighted and signal-to-noise parameters were chosen for the “enrichment statistics” and the “metric for ranking genes”, respectively.

Gene ontology (GO) enrichment analysis for the GO Biological Processes was performed by taking advantage of the Metascape tool (https://metascape.org; [77]). Pathway and process enrichment was performed.

### 4.4. Disease-Free Survival and Statistical Analysis

Spearman rank order correlation coefficients were computed between the normalized miRNA expression levels from the Hi-TCGA data set and the corresponding log2 RSEM-normalized counts of the putative targets. Statistical differences between groups were evaluated using the Welch *t*-test or a Mann–Whitney rank sum test, depending on the normal distribution of the variables considered. Tests were performed with R functions (R-3.6.2). Disease-free survival analyses were conducted considering updated clinical data (disease-free months and events) obtained from the cBioPortal (https://www.cbioportal.org/; accessed on 10 May 2023) for both TCGA and METABRIC cohorts. Patients were classified according to BRCA1 status and high or low levels of miR-29s, which were in turn determined by calculating the best cut-point based on the maximum log-rank value using the R package Survminer [78]. Survival was estimated using the Kaplan–Meier method, and differences between groups were evaluated with the log-rank test using the Survival and Survminer R packages [78,79].

## Data Availability

The data sets analyzed in the current study are publicly available from TCGA Research Network: TCGA-BRCA project, https://www.cancer.gov/tcga; from the METABRIC project (information at https://ega-archive.org/studies/EGAS00000000098); and from Gene Expression Omnibus database (GEO; https://www.ncbi.nlm.nih.gov/gds) for the GSE81002 and GSE59248 series.

## Supplementary Materials

The supporting information can be downloaded at: https://www.mdpi.com/article/10.3390/ijms24129916/s1.
