# Supplementary material for: In Silico Identification of a BRCA1:miR-29:DNMT3 Axis Involved in the Control of Hormone Receptors in BRCA1-Associated Breast Cancers
Source: Int J Mol Sci. 2023 Jun 8;24(12):9916. doi: 10.3390/ijms24129916 (PMC10298721; doi:10.3390/ijms24129916)
Supplement: Supplementary file 1 [file ijms-24-09916-s001.zip › Supplementary InformationNew.pdf]

Supplemental information

In silico identification of a BRCA1:miR-29:DNMT3 axis involved in the repression of hormone receptors in BRCA1-associated breast cancers.

Santarosa et al

.

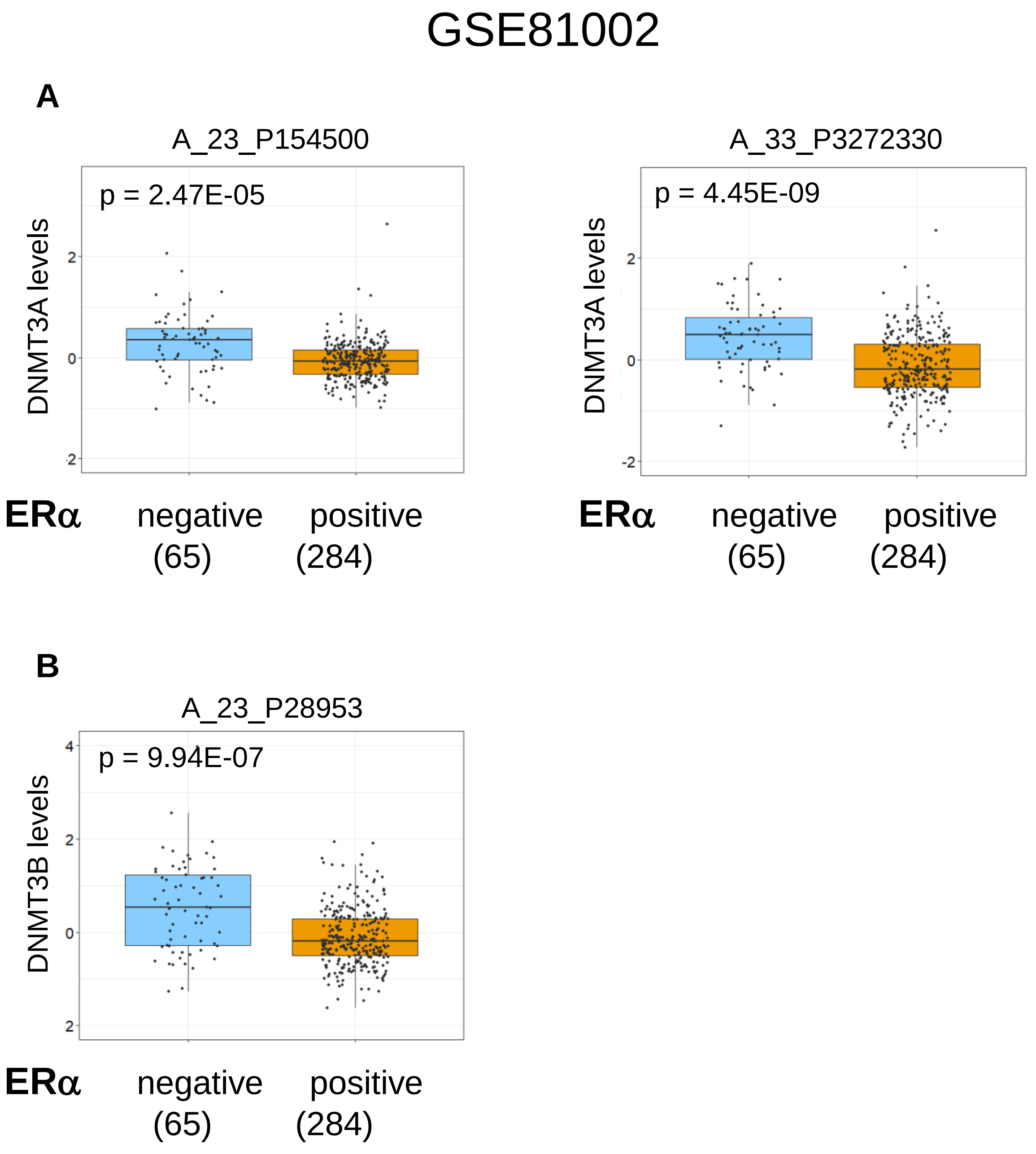

Supplemental Figure S1 **DNMT3A and DNMT3B levels according to hormone receptor status**

Data from GSE81002 show the expression levels of DNMT3A evaluated with two sets of probes (**A**) and the expression levels for the single probe of DNMT3B (**B**) in Estrogen Eeceptor ERα-positive and -negative samples. Progesterone receptor status was not provided. Statistical differences were determined by Welch t-test

GSE59248

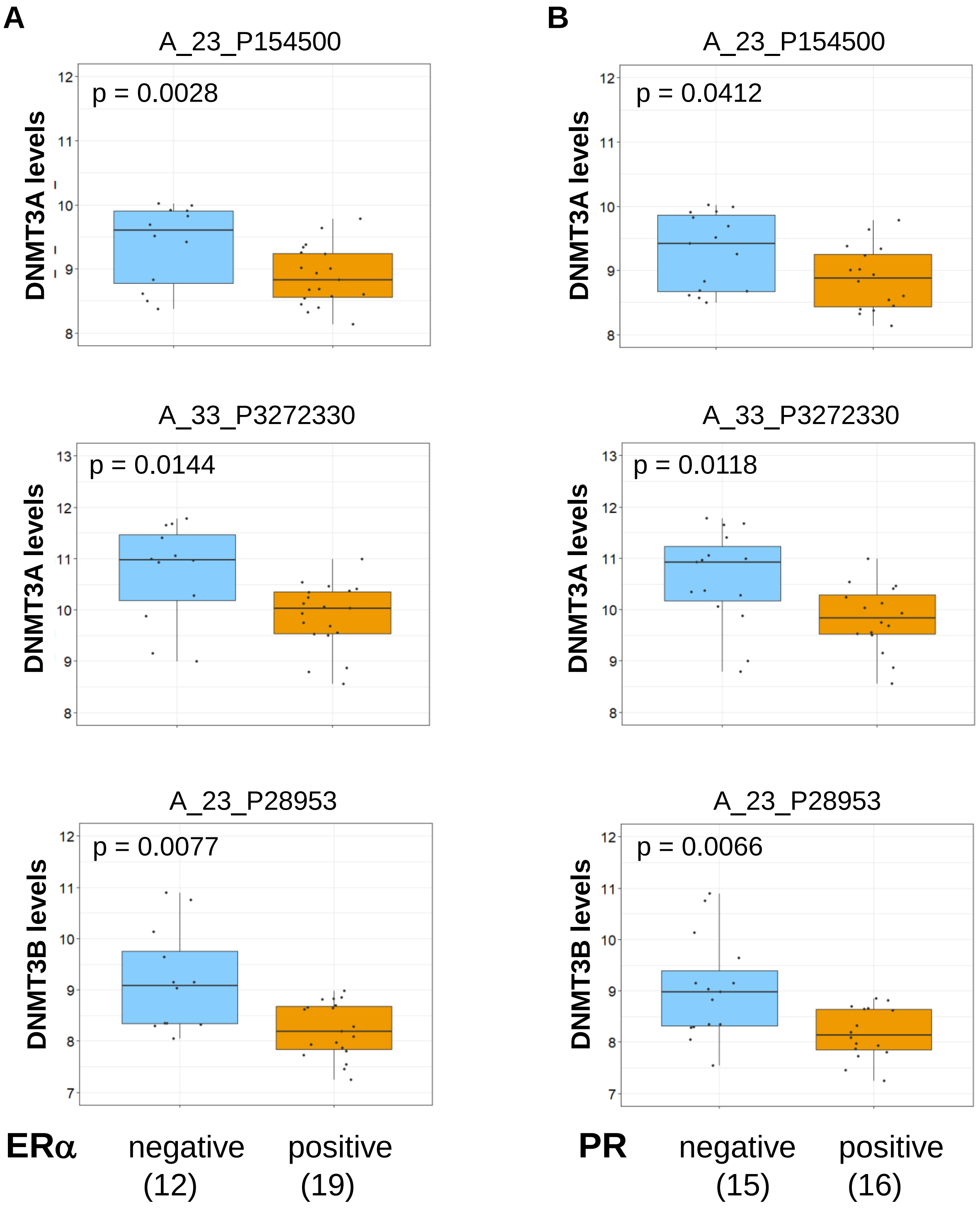

Supplemental Figure S2. **DNMT3A and DNMT3B levels according to hormone receptor status**

Data show the DNMT3A and DNMT3B levels in ERα-positive and -negative samples (**A**) and in Progesteron receptor PR-positive and -negative cases (**B**). Data from GSE59248 report the expression levels of two sets of probes for DNMT3A and one for DNMT3B. Statistical differences were determined by Welch t-test

ESR1

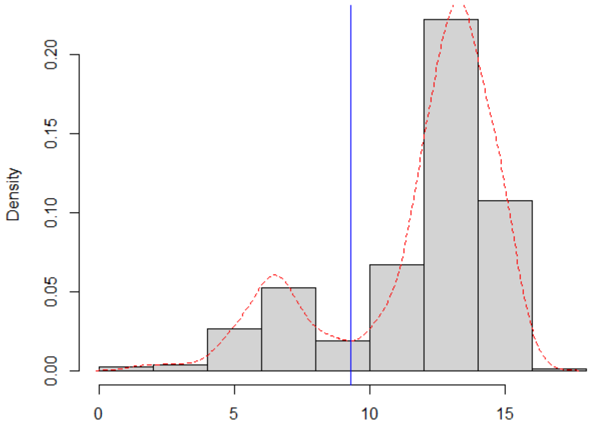

Gene	FC ESR1pos/neg	Mean_ESRneg	Mean_ESRpos	SD_ESRneg	SD_ESRpos	pvalues
ESR1	2.053	6.4404	13.2210	1.5410	1.2746	0.000

PGR

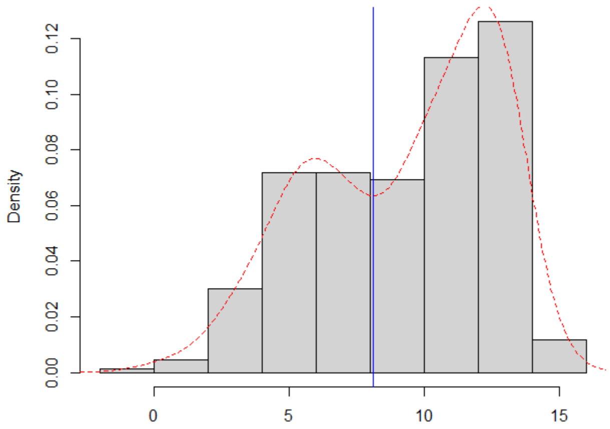

Gene	FC PGRpos/neg	Mean_PGRneg	Mean_PGRpos	SD_PGRneg	SD_PGRpos	pvalues
PGR	2.144	5.3797	11.5331	1.7340	1.6001	0.000

Supplemental Figure S3. **Density plots of the ESR1 and PGR transcripts** The graphs show the density distribution of the ESR1 (upper panel) and PGR (lower panel) transcripts (log2 RSEM-normalized counts) in Hi-TCGA series. The solid red curves represent the distribution. The blue lines represent the pit of the curves, whose corresponding transcript values are used as cutoff for positive and negative samples. Tables under the graph report differences in expression of ESR1 and PGR between positive (346 and 257 for ESR1 and PGR, respectively) and negative (87 and 157 samples for ESR1 and PGR, respectively) samples categorized as above.

Hi-TCGA

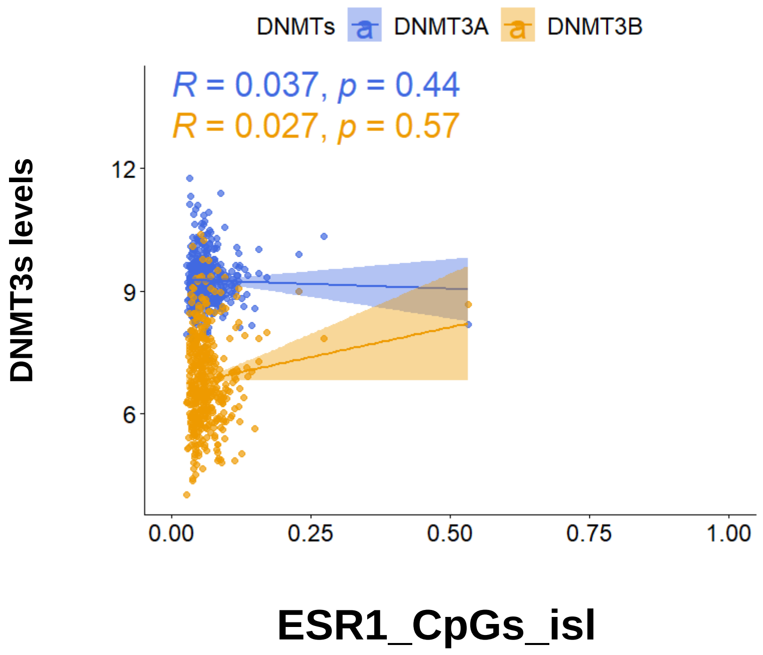

GA-TCGA

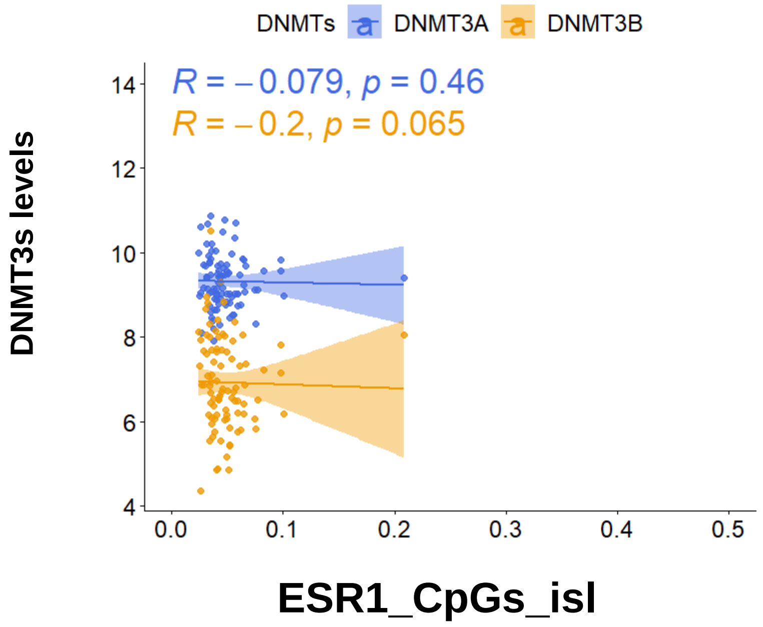

Supplemental Figure S4. **DNMT3A and DNMT3B levels and CpGs methylation**

Graphs shows the correlation (spearman coefficients) between DNMT3A (blue dots, lines, and data) and DNMT3B (orange dots, lines, and data) levels (log2RSEM normalized counts) and the mean of β-values of CpGs island in ESR1 gene in both Hi-TCGA (top panel) and GA-TCGA (botton panel) datasets.

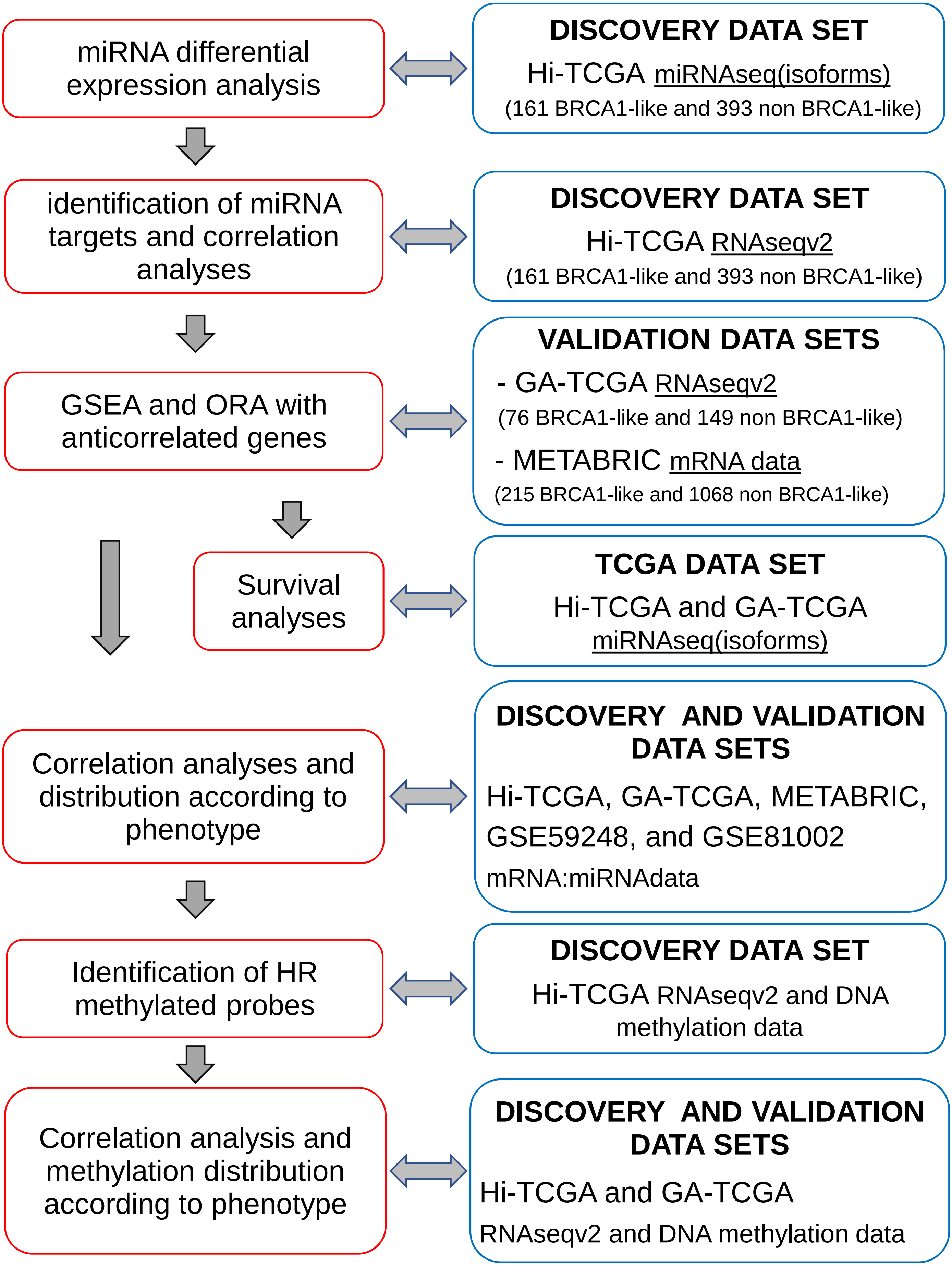

Supplemental Figure S5. **Schematic representation of the analyses performed and datasets used.** On the left (red edges) the analyses and on the right (blue edges) the datasets.
